# Continuum of Care Services for Maternal and Child Health using mobile technology – a health system strengthening strategy in low and middle income countries

**DOI:** 10.1186/s12911-016-0326-z

**Published:** 2016-07-07

**Authors:** Ramkrishnan Balakrishnan, Vijayaprasad Gopichandran, Sharadprakash Chaturvedi, Rahul Chatterjee, Tanmay Mahapatra, Indrajit Chaudhuri

**Affiliations:** CARE India, Patna, India; Department of Epidemiology, Fielding School of Public Health, University of California Los Angeles, Los Angeles, 90095 USA; Department of Community Medicine, ESIC Medical College & PGIMSR, KK Nagar, Chennai, 600078 India

**Keywords:** mHealth, Maternal and child health, Health system, Efficiency, Quality

## Abstract

**Background:**

Mobile phone technology is utilized for better delivery of health services worldwide. In low-and-middle income countries mobile phones are now ubiquitous. Thus leveraging mHealth applications in health sector is becoming popular rapidly in these countries. To assess the effectiveness of the Continuum of Care Services (CCS) mHealth platform in terms of strengthening the delivery of maternal and child health (MCH) services in a district in Bihar, a resource-poor state in India.

**Methods:**

The CommCare mHealth platform was customized to CCS as one of the innovations under a project funded by the Bill and Melinda Gates Foundation to improve the maternal and newborn health services in Bihar. The intervention was rolled out in one project district in Bihar, during July 2012. More than 550 frontline workers out of a total of 3000 including Accredited Social Health Activists, Anganwadi Workers, Auxilliary Nurse Midwives and Lady Health Supervisors were trained to use the mHealth platform. The service delivery components namely early registration of pregnant women, three antenatal visits, tetanus toxoid immunization of the mother, iron and folic acid tablet supply, institutional delivery, postnatal home visits and early initiation of breastfeeding were used as indicators for good quality services. The resultant coverage of these services in the implementation area was compared with rest of Bihar and previous year statistics of the same area. The time lag between delivery of a service and its record capture in the maternal and child tracking system (MCTS) database was computed in a random sample of 16,000 beneficiaries. The coverage of services among marginalized and non-marginalized castes was compared to indicate equity of service delivery. Health system strengthening was viewed from the angle of coverage, quality, equity and efficiency of services.

**Results:**

The implementation blocks had higher coverage of all the eight indicator services compared to rest of Bihar and the previous year. There was equity of services across castes for all the indicators. Timely capture of data was also ensured compared to paper-based reporting.

**Conclusion:**

By virtue of its impact on quality, efficiency and equity of service delivery, health care manpower efficiency and governance, the mHealth inclusion at service provision level can be one of the potential strategy to strengthen the health system.

## Background

### mHealth technology

With the ubiquitous proliferation of mobile phone in low-and-middle income countries (LMIC), communication technology has expanded in its scope and potential uses [1]. mHealth technology is the use of mobile phones and wireless communication techniques for achievement of health goals. Mobile communication technology could be put to various applications in health [2]. Mobile technology has been commonly used in serving as health call centres, as toll free emergency contact numbers, for managing disaster and emergency situations and for providing mobile telemedicine [2]. Other common uses of mHealth technology include decision support, reminders for health care visits, monitoring of health care programs and effective service delivery [3]. In 2009, the second global eHealth survey was conducted by the World Health Organization among the 112 member countries [4]. It was found that mHealth is emerging as an important health intervention in most member countries.

### Effectiveness of mHealth technology in improving health systems

Mobile based applications have been adapted in several disease conditions and have demonstrated effectiveness. A recent scoping review showed that mobile technology contributed to reduction in delays in accessing maternal health in LMIC [4]. Yet another systematic review demonstrated the effectiveness of mHealth in improving access, coverage and equity gaps in delivery of chronic disease services in LMIC [5]. A recent systematic review concluded that use of mobile technology as a health provider decision support system has modest benefits. However when the mobile camera photos were used for diagnosis a reduction in correct diagnosis was observed [6].

A review of mHealth innovations in LMICs showed that there is dearth of evidence to demonstrate effectiveness of these interventions on clinical outcomes. There are very few mHealth interventions which have focused on community health workers. These included data collection intervention, SMS based reminders for patients, job aids for health workers and decision support mechanisms [7, 8].

A cluster randomized controlled trial of SMS based text messaging about appropriate treatment for malaria in Kenya revealed a significant 23 % improvement in appropriate treatment in the intervention arm [9]. A mixed methods evaluation of mHealth intervention in rural Uganda for AIDS control revealed that there was no quantitative difference between the intervention and control groups, however qualitatively reported perceived improvements in patients care, logistics and support [10]. Use of personal digital assistants (PDA) in health related data capture in Uganda showed a 91 % perceived benefit for each unit of spending [11].

USAID in collaboration with World Vision implemented a mHealth intervention in Afghanistan in the field of maternal and child health and demonstrated effectiveness of the intervention in some maternal and child health outcomes [12].

In a study comparing paper based and mobile based monitoring and evaluation of community health worker activities showed that the mobile based system significantly improved data transfer accuracy compared to the paper based system [13]. Several studies have shown the effectiveness of mHealth technologies in advancing maternal and child health care in low and middle income countries [14–16].

Though there is enough evidence as reported in the previous paragraphs to support effectiveness of vertical disease specific interventions using mHealth technology, little is known about the role of mHealth in strengthening health systems.

### Health system strengthening

The World Health Organization defines health system as “consisting of all organizations, people and actions whose primary intent is to promote, restore or maintain health in ways that are responsive, financially fair and make the best or most efficient use of available resources”. The ultimate goal of the health system is to improve the health of the community [17]. People are at the center of this system [18]. This includes the community, who are the beneficiaries of the system. It is the people’s resources that are utilized towards delivery of these services. The health system is described as a complex adaptive system. A complex adaptive system, as the name indicates is ‘complex’, involving a variety of components, ‘adaptive’, each component has an independent ability to adapt and change and is a ‘system’, a set of connected and interdependent parts [19, 20]. The independent parts of a complex adaptive system are capable of their own autonomous function, operate within their own environmental conditions and responses, interact with other agents and keeps evolving based on their interactions [19].

All interventions in health systems have system-wide effects. For example, if there is a health worker training intervention, its effects are not just limited to their efficiency alone, but involve all other components such as service delivery, resource allocation, etc. Many health interventions are not individual component level interventions but are system-wide. Health worker training is one such system-wide intervention. Thus systems level thinking will consider not only the individual components of the system but also how the components act, react and interact with each other [21, 22].

### mHealth as a health system strengthening strategy

In order to assess whether mHealth innovations strengthen health systems, as described previously, systems thinking should be adopted. mHealth innovations should be viewed as a component of the complex health system. Coverage, quality, equity and efficiency in achieving the outcomes of good health, financial risk protection and responsiveness will ensure strengthening of the health system. The mHealth intervention cannot be seen as a standalone one. It should be viewed as integrated into the system.

Though there have been several studies reporting the effectiveness of mHealth systems and several case reports of successful mHealth applications, there are very few evaluations of mHealth application as a health system strengthening strategy [23]. This study reports an mHealth application implemented in Bihar, India as a health system strengthening strategy and views it from a health systems angle.

## Methods

### Continuum of care services (Maternal and Child) using mHealth

**Fig. 1:**
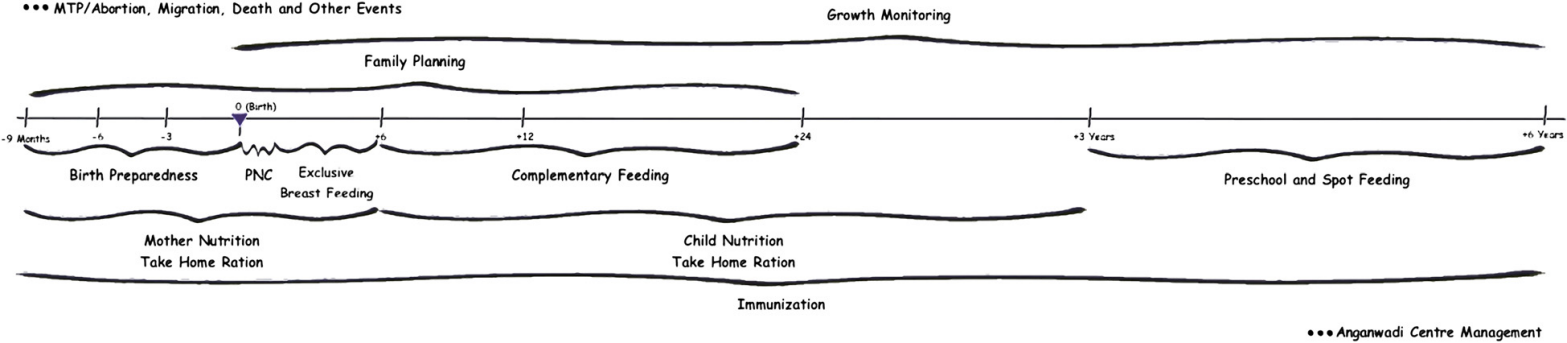
Continuum of Care Services for Maternal and Child Health from registration of pregnancy to the child’s 6^th^ year of age

A public health program to improve maternal and newborn health was initiated in Bihar by Care India with the support of Bill and Melinda Gates Foundation with the overall goal of achieving the Millennium Development Goals (MDG) 4 and 5. As an innovation in this program, the Continuum of Care Services (CCS) - (Maternal and Child) was introduced using an mHealth platform. The mHealth platform is used for case management by the frontline community health workers (FLW). Under the CCS intervention pregnant women or newborns will be entered as a case into the system and followed up and care provided till the child is 6 years old (name based tracking of each pregnant mother and child in the FLW service area). There are specific units in the CCS mHealth application for pregnancy registration, antenatal care, birth preparedness, delivery, post natal care, exclusive breastfeeding, immunization and complementary feeding and growth monitoring. This CCS is depicted in Fig. 1.

CommCare is a technically advanced, evidence based mobile platform of Dimagi which is available for use in low resource settings for mobile applications including mHealth. Dimagi developed the CCS application based on the CommCare platform with specific inputs on content and processes from the expert team of Care India. The CCS application has a home visit planner for the frontline worker. It also has a built in scheduler and a checklist which helps the frontline worker perform her activities seamlessly and at the same time collects data from the field for real time transmission to the central database. Different multimedia job aids are also integrated into the CCS platform to help inter personal communication by the frontline worker. The central database also keeps the data of the client synchronized between the mobile phone, the supervisor’s mobile and the central database which helps in monitoring and supportive supervision. In India, maternal and child health care services are delivered by two systems namely the Ministry of Health and Family Welfare (MoHFW) and the Ministry of Women and Child Development (MoWCD). The frontline worker for the former is the Accredited Social Health Activist (ASHA) and the latter is the Anganwadi Worker (AWW). The CCS platform had a provision to integrate the work of these two frontline workers. The end user of the application was the Front Line Workers (FLWs). They were provided mobile phones with the pre-installed application. The complete details of the software program that were existing in the CommCare mobile platform and the unique features of the CCS program are shown in Table 1.

**Table 1:** Features of the existing CommCare mobile platform and new features added in the CCS platform

Features in CommCare mobile platform	Features added for CCS mobile platform
Form Creation: Form builder Interface or upload XLS form	Registration and name based tracking of beneficiaries – pregnant women and children below 6 years of age
Workforce Management: Creating, managing and monitoring frontline workers and their day-to-day application usage	Automatic scheduling of home visits for the frontline workers
Case Management: Creating a case and tracking through various forms	Automatic generation of list of children due for immunization
Multimedia Integration: Images, Audio, Video	Interactive checklist and counseling protocols to promote and gather maternal and child health in the continuum
Language: Local Language Support	Animated videos as job aids for providing inter personal communication
Logics and Calculations	Tools to compute expected date of delivery, body mass index, child growth standards
Reports: Basic interface reports and via email configuring	Convergence and case sharing between health (MoHFW) and nutrition (MoWCD) departments at service delivery level
Export Formats: XLS, CSV, ZIP	Data driven supportive supervision for periodical review and monitoring
Messaging Compatibility: SMS, IVR	Synchronization of frontline worker activities with supervisor to enable indicators based monitoring through CloudCare
Application Programming Interface (API) Access	Activities based approach and effortless data recording of each beneficiary in the continuum

The intervention was rolled out in the Saharsa District of Bihar in eastern part of India in the June 2012 and is ongoing. The data collection for the analysis was stopped in March 2015. Saharsa is one of the 38 districts of Bihar. The total population of the district is 1.8 million. The population is predominantly rural. The sex ratio is 906 females per 1000 males and the literacy rate is 54.57 %. In 2006, the district was named among 250 most backward districts of India and in 2013 government of India identified the district as one of the High Priority District (HPD) to promote Reproductive, Maternal, Newborn, Child and Adolescent health (RMNCH + A) The district has some of the lowest achievements in terms of health indicators in Bihar. This analysis is part of a larger implementation study that was conducted in this area which looked at comparative efficiency of the mHealth intervention in select blocks of this district versus paper based routine service delivery in other blocks.

### Analysis plan

**Fig. 2:**
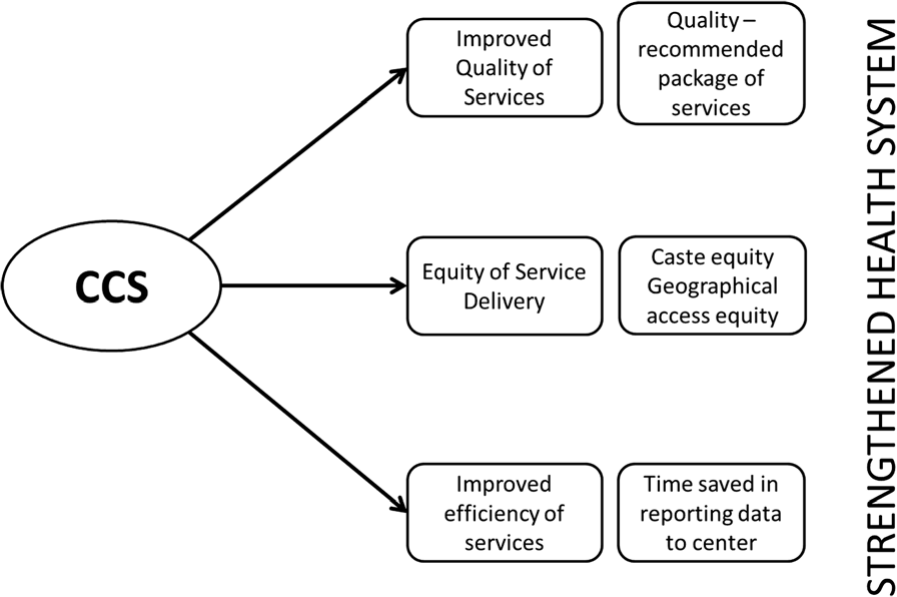
Conceptual model for the study of CCS as a health system strengthening strategy

The conceptual framework employed for this study is shown in Fig. 2.

The effect of the mHealth CCS intervention on improvement of quality, equity and efficiency of services was studied. When the mother and child received appropriate package of services including early registration of pregnancy, three antenatal visits, tetanus toxoid injections, iron and folic acid supplementation, institutional delivery, early initiation of breastfeeding and postnatal home visits, the quality of service was said to be good. This was measured among the women who were eligible for these services in the district where the CCS was rolled out and compared with the regular maternal and child health service delivery data of the rest of the state of Bihar. In order to assess equity of service delivery, these key indicators were compared between various castes (scheduled castes and tribes) in the CCS area. Finally the time gap between occurrence of a specific event (like vaccination of a child) and its reporting in the regular maternal and child service database of the state of Bihar was documented as an indicator of time efficiency.

The study data were tabulated from two main sources namely, the Maternal and Child Tracking System (MCTS) database of the National Rural Health Mission for the whole of Bihar and the data captured through the mHealth CCS platform for the implementation project district. The tabulated data was summarized and compared.

The study protocol and procedures were reviewed and approved by the Institutional Committee for Ethics and Review of Research (ICERR) of the Indian Institute of Health Management Research (IIHMR), Jaipur, India. Written informed consent was obtained from all the participants in the intervention. Data confidentiality was ensured by restricting access to the data to those who needed to know.

## Results

The details of the implementation of the CCS project are given in Table 2. A total of 512 Frontline workers including ASHAs and AWWs were trained for implementation of the CCS project. Training was provided to the frontline workers through an incremental learning process. Each FLW underwent 4 h of training and practice sessions per week for 8 consecutive weeks through facilitators on the mobile application as well as provision of maternal and child health care. Additional supportive supervision was made available during the intervention at the field level. Between the period of 1^st^ July 2012 and 10^th^ March 2015 a total of 19,880 pregnancies and 19,888 children were registered and provided the continuum of care services. A total of 3,09,733 home visits were provided for these women and children.

**Table 2:** Details of CCS project implementation in the district

S. No	Implementation characteristic	Numbers
1	Frontline workers trained	512
2	Supervisors trained	58
3	Total number of pregnant women registered	19,880
4	Total number of children registered	19,888
5	Total number of home visits made by frontline workers	3, 09,733

Data for the period of July 2012 to March 2015

### Quality of continuum of care services

Good quality continuum of care services was defined using the previously described eight indicators which are essential for a good maternal and child health. Table 3 shows the coverage of each of these indicators in the implementation area for the period of July 2012 to August 2014 and compares it with the indicators for the state of Bihar for the period between April 2012 to April 2013 obtained from the Maternal and Child Tracking System database.

**Table 3:** Indicators of coverage of continuum of maternal and child care services in implementation district

S. No	Indicator	Coverage in implementation area (95 % CI) ^a^	Coverage in implementation District in previous year (%) ^b^	Coverage in rest of Bihar (%) ^c^
1	Registration of pregnancy	83.03 (78.12–86.30)	56.30	50
2	Registration within the first trimester	15.46 (10.54–19.20)	10.11	10
3	Complete 3 antenatal visits	55.94 (48.40–59.23)	50.55	48
4	Received at least one TT vaccine	79.38 (58.90–80.26)	74.12	80
5	Received more than 90 Iron and Folic Acid Tablets	61.59 (44.3–68.4)	50.23	49
6	Delivered in a health facility	83.93 (73.45–88.36)	58.90	66.5
7	Baby breastfeeding initiated within 1 h of birth	97.72 (80.23–98.67)	73.45	73
8	Received at least one post-natal home visit	28.41 (12.45–42.56)	18	10

^a^ Data from July 2012 to Aug 2014 ^b^ Data from Apr 2011 to Apr 2012 ^c^ Data from Apr 2012 to Apr 2013

It is seen that registration of pregnancy, early registration, complete 3 antenatal visits, receiving at least 90 iron and folic acid tablets, institutional delivery, early initiation of breastfeeding and post natal home visits are all higher in the implementation area compared to rest of Bihar during the same time. It is also seen that these indicators are better in the same district compared to the previous year.

### Equity of service delivery

**Fig. 3:**
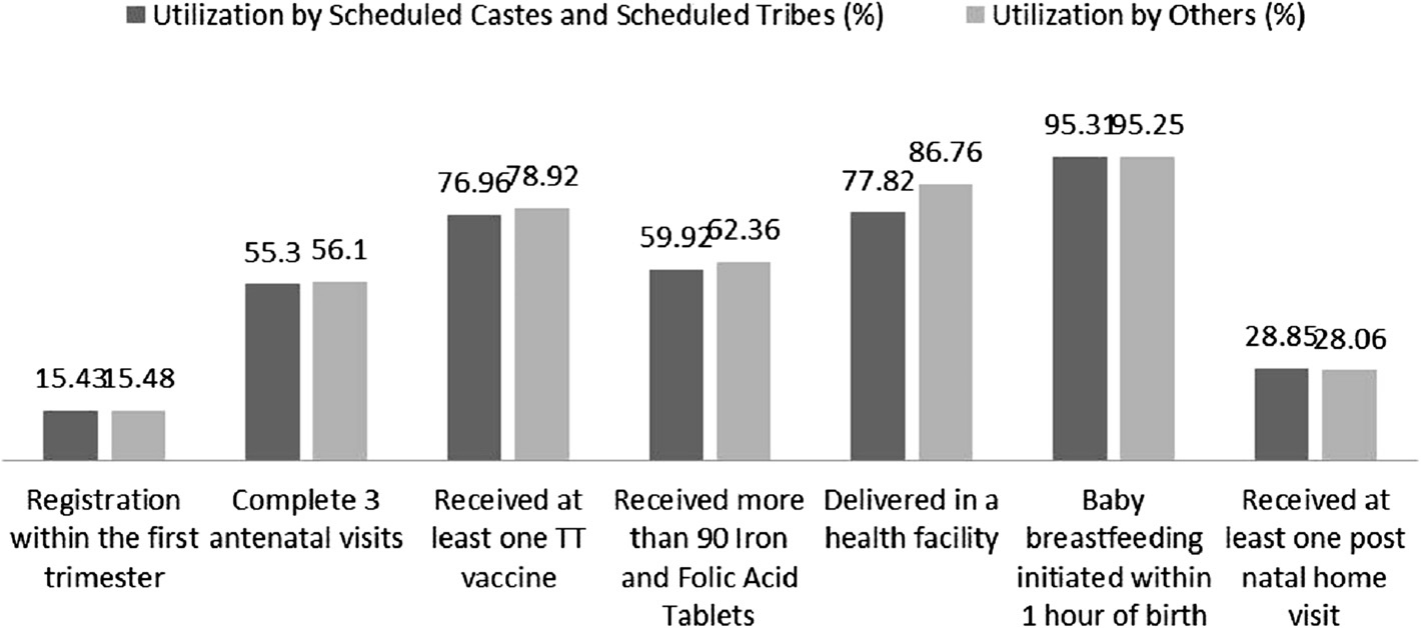
Caste equity in utilization of continuum of maternal and child care services in the implementation district

In order to assess equity of services caste wise disaggregated data of coverage of services was assessed. This is shown in Fig. 3.

The continuum of care services were delivered equitably to scheduled castes/tribes and the other castes in all aspects. Only institutional deliveries were higher among the other castes compared to the scheduled castes and tribes.

### Efficiency of services

Table 4 shows the time delay between delivery of service and data capture in the maternal and child tracking system database.

**Table 4:** Time lag in data transfer from health worker records to maternal and child tracking system (MCTS) database using the paper method

Description	Time delay/Proportion
Average time between service delivery and data capture in MCTS	72 days
Longest time between service delivery and data capture in MCTS	1113 days
Shortest time between service delivery and data capture in MCTS	0 days
Proportion of data entered into MCTS on the same day of service delivery	0.04 %

The average time lag of 72 days which is approximately two and half months, is overcome by the instant data capture that is possible with the mHealth CCS platform.

## Discussion

This study evaluated the overall feasibility and efficacy of using mHealth technology for delivering the continuum of maternal and child care services in one district of the state of Bihar. In this paper we view the CCS mHealth platform as a health system strengthening strategy from the angle of improved quality, equity and efficiency of service delivery. The analysis found that the coverage of eight key indicators of the continuum of maternal and child care services were higher in the implementation block compared to rest of Bihar and the previous year’s data (before implementation of the mHealth intervention). However the increase in Iron and Folic Acid uptake was not very high given the person interaction and inputs from the FLWs. This is probably because the supply of Iron and Folic Acid tablets is a job of the Auxilliary Nurse Midwife and not these FLWs. Moreover, there was also a shortage of supply of Iron and Folic Acid Tablets in the intervention blocks during the intervention period. Similarly receiving at least one post natal visit is also not adequately improved in the intervention areas. The reasons for poor improvements in post natal visit has been challenging and needs further exploration, which was beyond the scope of this study. It was also found that the implementation of CCS lead to equitable reach of services and the data capture was more time efficient. From all these three angles, the implementation of CCS led to strengthening of the health system in the district.

The better coverage of all eight indicators in the implementation period could indicate either better service delivery or better reporting of services. In order to verify this, it is important to look at a concurrently done household survey in the implementation area and a control area where the traditional services were provided and paper based reporting was carried out. In the household survey evaluation it was seen that the beneficiaries reported greater number of home visits in the antenatal period, within 24 h of delivery, within 1 week of delivery, and a complementary feeding home visit in the intervention villages compared to control villages. About 50 % of the beneficiaries in the intervention group and 29 % of beneficiaries in the control group received all three antenatal visits as reported in the household survey. Early initiation of breastfeeding was reported to be higher in the intervention group (76 %) compared to the control group (62 %). About 41 % of the children in the intervention group were initiated on complementary feeding at an appropriate time as against 32 % in the control group [24]. Thus we can conclude that the findings are not just improved reporting but actual improvements in service delivery.

In a previous USAID supported mHealth intervention in Afghanistan, the use of mobile technology by community health workers led to significant improvement in knowledge of women about maternal and child health. It also led to better health seeking behaviors. When the mobile was used as a job aid it increased the participation of women in the counseling sessions led by the health workers. The mobile phone also increased coordination of referral services and delivery of medical supplies [12]. Similarly a previous study has also shown that effective use of mHealth platforms can improve data capture efficiency and accuracy. The mHealth system also ensured a longitudinal follow up of each referral by back referral of patients. The study also emphasized the need for close supportive supervision of both paper based and mHealth system of monitoring and evaluation [13].

In this paper we look at the mHealth intervention from a health system perspective. When the health system, comprised of the six essential building blocks namely service delivery, information and research, medical products and technologies, health workforce, health care financing and leadership and good governance, operate with good coverage, access, quality and safety it leads to good health outcomes. These good health outcomes could be tangible improvements in health indicators, responsiveness, financial risk protection and improved efficiency [14]. The CCS mHealth intervention improves service delivery, information capture, health workforce efficiency, good governance and also improves coverage, access, quality and equity as demonstrated in this study. By improving all these components it may be said to strengthen the health system.

There are several limitations in this evaluation. Firstly this is a quasi-experimental evaluation with its own limitations. The data from the mHealth district is compared with data from rest of Bihar as well as historical data of the previous year. This could lead to potential confounding effect in analysis. Since raw data were not accessible from the maternal and child tracking system and only summarized data was used in the analysis, fixed or random effects modeling could not be performed to assess the effects of this limitation. A more robust evaluation and analysis of the mHealth intervention and Maternal and Child Tracking Systems will reveal the unbiased effect size of benefits due to the intervention. This paper does not report the actual reasons for why certain outcome measures did not improve substantially following the intervention i.e., iron and folic acid uptake. However more detailed discussion of this intervention can be found in the report of the larger controlled study of the mHealth platform reported in this paper [24]. Future studies should be conducted as a cluster randomized trials to have a good contemporary comparison group. Such research studies will help establish the efficacy of the intervention clearly.

## Conclusion

The CCS mHealth intervention to help frontline workers delivery maternal and child health services in a backward district in eastern India showed significant improvements in quality of service delivery, reporting of service delivery, time efficiency of information capture and equity in delivery of services. By virtue of its impact on quality, efficiency and equity in service delivery, health care manpower efficiency and governance the mHealth intervention can be said to strengthen the health system. This project demonstrates the importance of the role of mHealth in effective health care service delivery in low and middle income countries.
